# Does One Treatment Fit All? Effectiveness of a Multicomponent Cognitive Behavioral Therapy Program in Data-Driven Subtypes of Perinatal Depression

**DOI:** 10.3389/fpsyt.2021.736790

**Published:** 2021-11-17

**Authors:** Ahmed Waqas, Atif Rahman

**Affiliations:** Department of Primary Care & Mental Health, Institute of Population Health, University of Liverpool, Liverpool, United Kingdom

**Keywords:** perinatal depression, heterogeneity, disability, subtypes, machine learning, cluster analysis, postpartum depression, clinical phenotypes

## Abstract

**Background:** Current diagnostic systems of mental disorders are criticized for their poor validity and reliability, owing to the within disorder heterogeneity and between disorder homogeneity. The issue is important if treatments for mental disorders are to be tailored to individual needs. There is little information in this area on perinatal depression (PND), a highly prevalent condition globally.

**Aims:** i) Quantify heterogeneity attributable to the polythetic diagnostic framework for PND and, ii) present evidence for the effectiveness of a multicomponent and low-intensity cognitive behavioral Thinking Healthy Programme (THP) across the heterogeneous presentations of PND.

**Methods:** This investigation presents secondary analyses of a cluster randomized controlled trial, conducted in Kallar Syedan, Pakistan. A total of 903 pregnant women were randomized to an intervention group receiving the THP intervention or control group receiving enhanced usual care. Principal component analyses and clustering algorithm were utilized to identify heterogenous subtypes of PND. Linear mixed effects models were used to assess effectiveness of the intervention across the identified subtypes of PND.

**Results:** Four different clusters of PND were identified: mixed anxiety-depression, somatic depression, mild depression, and atypical depression. All clinical phenotypes responded well to the THP intervention. Compared to their counterparts in the control group, mothers with mild depression in the treatment group yielded lowest risk ratios 0.24 (95% CI: 0.15 to 0.37), followed by mothers with anxiety-depression 0.50 (95% CI: 0.37 to 0.68), atypical depression 0.51 (95% CI: 0.27 to 0.99) and somatic depression 0.59 (95% CI: 0.42 to 0.83).

**Conclusion:** The Thinking Healthy Programme was found to be effective in reducing severity of depressive symptoms and disability across the four subtypes of PND.

## Background

### Perinatal Depression

While research trends in quantifying heterogeneity in mental disorders have gained momentum, knowledge gaps exist in perinatal psychiatry. The present investigation thus, aims to explore heterogeneity in perinatal depression (PND). PND is a prevalent and debilitating common mental disorder associated with pregnancy and child-birth. Although common worldwide, women living in low- and middle-income countries such as Pakistan report a higher preponderance toward the condition (1, 2). The high prevalence of PND is associated with deleterious effects on psychosocial functioning of the mothers and child development (1, 3). Previous PND literature has shown that the PND is a complex disorder with a biopsychosocial etiology, compounded by social risk factors including stressful birthing experiences and role transition to motherhood (2). Limited research on biological underpinning of PND shows dysregulation of Hypothalamic Pituitary Adrenal (HPA) axis, oxytocin system, inflammatory response, neuronal differentiation and circadian rhythms (4, 5).

### Heterogeneity in Perinatal Depression

Symptom-wise, PND presentation is idiosyncratic with a complex and yet poorly understood gene-environment interaction (6). This complex nature of PND is likely to result in a highly heterogeneous disorder, with varying onset, longitudinal trajectory, and symptom configuration (6). This variability in PND among women may hamper the assessment and diagnosis of this condition, and result in variable response to prevention and treatment strategies. Therefore, an understanding of the heterogeneity for PND ought to be emphasized for development of personalized prevention and treatment. Despite this recognition, there has been little research on this aspect of PND, with previous literature targeted on major depressive disorder.

With the recent advent of powerful statistical and machine learning procedures, it has become possible to study the heterogeneous nature of PND (6–8). Traditionally, the categorical conceptualization of mental disorders was done clinically, as evident in the Diagnostic & Statistical Manual and the International Classification of Diseases. This approach was also evident in the field of psychometrics, where a mental disorder was assessed as a latent construct based on a constellation of symptoms (9). This was usually done using a variety of rating scales that classify depression as a categorical diagnosis (yes/no) or symptom severity levels (mild/moderate/severe) (10–12). Although these approaches have improved our understanding of mental disorders especially depression, these problematic assumptions have limited it as an essentially homogenous disorder, hampering our understanding of causation and mechanistic mediational pathways (13). The limited understanding of the condition might also translate to a lack of efficacious and tailored interventions that work across specific contexts. Epidemiological, genetic and intervention studies rely on psychiatric diagnoses, which, if inadequate, might lead to erroneous conclusions (9, 14, 15).

### Objectives of the Study

Unfortunately, while the evidence for the differences in MDD subtypes is available, it is virtually non-existent for PND, a subtype of depression associated with pregnancy. This subtype of depression has been identified by the DSM and ICD as an individual identity and PND is a distinct condition with a different pathophysiology when compared with MDD among other study populations (12). Therefore, this study explores two research objectives:

- Present evidence pertaining to heterogeneous combination/constellation of PND symptoms among perinatal women in Pakistan and explore subtypes of PND, driven from exploratory analyses and unsupervised machine learning techniques.
- Present evidence pertaining to effectiveness of the multicomponent Thinking Healthy Programme across the newly identified subtypes of PND.

## Methods

### Study Design

The present investigation uses data from the baseline assessments of a large-scale cluster randomized controlled trial (cRCT) assessing the effectiveness of a cognitive behavior therapy-based intervention by community health workers for perinatal depression (16). Briefly, this cRCT was conducted in two rural subdistricts of rural Punjab: Gujar Khan and Kallar Syedan, with a combined population of about 750,000 people, and comprising of 40 union councils (administrative units). The union councils also served as the cluster unit of randomization. These rural areas are geographically, ethnically, and socioeconomically homogenous and comprise primarily of low to lower middle class rural households.

Both subdistricts are served by basic health units (BHU) that deliver primary healthcare to the residents. In addition to doctor and a midwife, each BHU is also staffed by 15 to 20 lady health workers. These women are community health workers that provide preventive maternal and child health services in the area. For data collection and delivery of intervention, LHWs in each union council were trained in conduct of baseline and follow up assessments as well as delivery agents. Further details on the study design of the cRCT have been reported in the primary publication (16). Ethical approval for the original randomized controlled trial was granted by the University of Manchester, UK and the Institute of Psychiatry, Rawalpindi, Pakistan. The present secondary analyses, however, were exempt from ethical review. Informed verbal and written consent were taken the participants, who were assured anonymity and that only group level findings will be reported.

For recruitment, LHWs in their union councils invited eligible participants identified using a central official register maintained at each BHU. Study participants were pregnant women in their third trimester, aged 16 to 45 years and married. Exclusion criteria included diagnosis of a serious medical condition, physical or learning disability or severe psychiatric illnesses. The baseline assessments were conducted by two experienced psychiatrists, from April 2005 to March 2006. Using the structured clinical interview for Diagnostic and Statistical Manual of Mental Disorders (DSM)-IV diagnosis of perinatal depression, all eligible women fulfilling the criteria of PND were recruited in the trial. Post-intervention and follow up assessments were conducted at 6 months and 12 months, respectively (17). Thereafter, long term follow-up was conducted for the mother-child dyads when the index child was 7 years old.

### Intervention

Thinking Healthy Programme (THP) is a multicomponent cognitive behavioral therapy based intervention that was designed primarily as a task shifted intervention (18). The standard THP is an evidence-based manualised intervention targeting women with perinatal depression in low socioeconomic settings (18). It aims to improve health outcomes among mothers and their children through the adaptation and integration of Cognitive Behavior Therapy (CBT) techniques into the routine work of LHWs (18). Non-specific techniques include active listening, psychoeducation and fostering social support from key family members to support the mother in negotiating the challenges she faces. Owing to its effectiveness and ease of delivery, it was recommended by the World Health Organization as a model intervention and included in the Mental Health GAP Action Programme (mhGAP) (19) for scaling up of mental health services around the world. THP trains non-specialist delivery agents in several evidence-based strategies to improve symptoms of perinatal depression.

The CBT techniques include challenging unhelpful thoughts, behavioral activation, and problem solving. It includes cognitive behavioral therapy sessions delivered in a simplified form through pictures, illustrations, and activities. The THP illustrates the use of CBT strategies in three simplified steps. By employing guided discovery technique, LHWs use culturally appropriate figures and illustrations to educate the intervention recipients about maladaptive thinking styles and ways to identify them. Intervention recipients are educated about strategies to replace maladaptive thoughts and use alternative healthy strategies. Thereafter, they are educated about the link between thoughts and actions. They are further taught the use of health calendar to monitor their thoughts and behaviors to practice healthy thinking.

All modules further provide psychoeducation to mothers using commonly used terms for mental health to avoid stigma. It also aims to improve mothers' social support networks by teaching them strategies to identify and acquire social support from people around her and encourages engagement with delivery agents. Finally, advice on mother-baby relationship and practical activities aim to improve postnatal bonding and physical health and nutrition of the baby. It also encourages adjunct techniques such as physical activity, relaxation and problem solving (20). The intervention thus aims to improve maternal well-being, mother-infant interaction and relationship with key family members including spouses. The intervention identifies resources within the family and community to address potential risks to her mental health (20).

**Figure 1:**
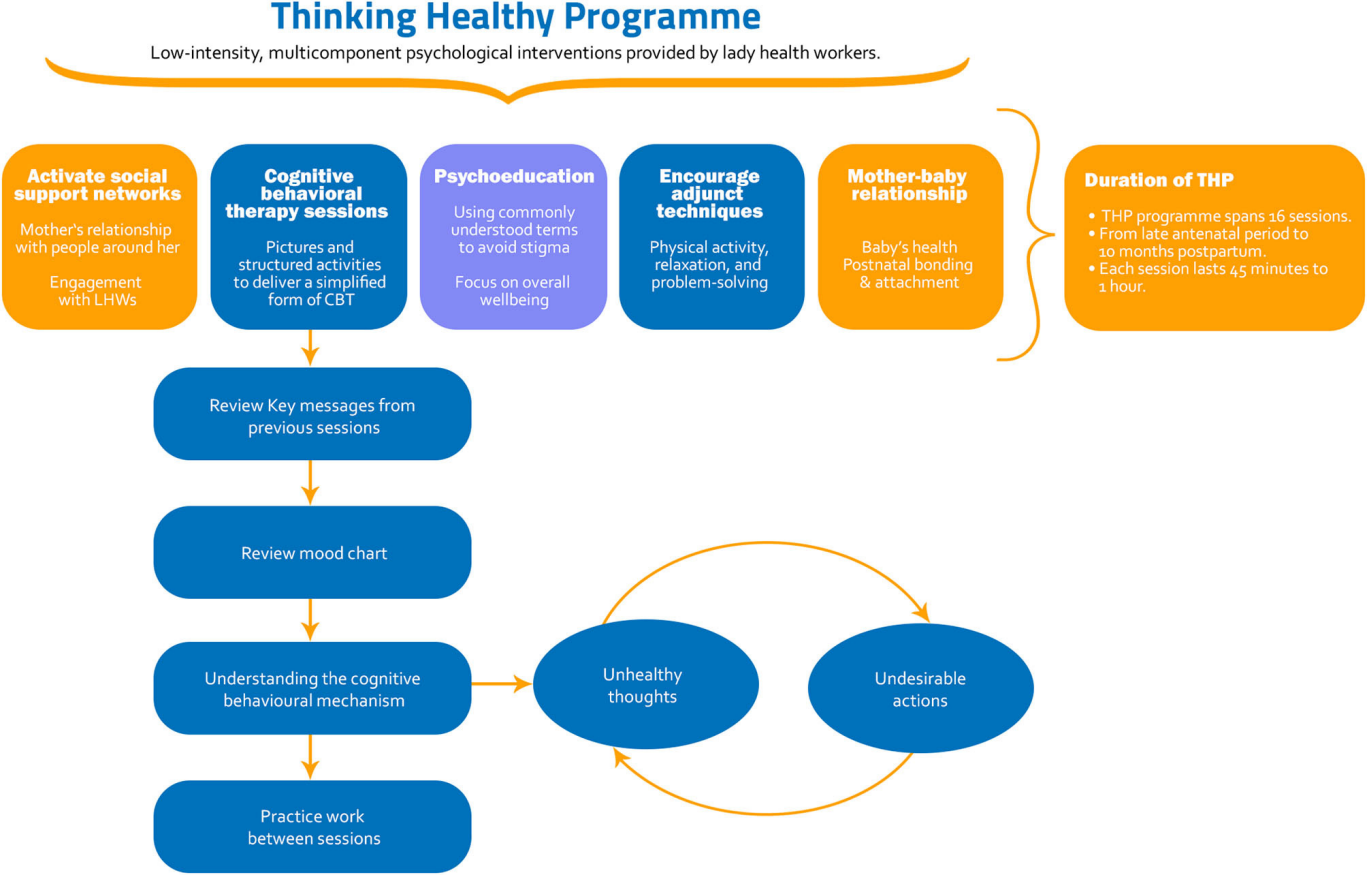
Content of the thinking healthy programme.

The intervention programme comprised of a total of sixteen session. After one or two introductory sessions with the LHW, one session of the THP was delivered weekly for four weeks in the last month of pregnancy. These sessions comprise the module 1 of the THP, called the “preparing for the baby”. This was followed by three sessions in the first month postpartum, collectively termed as module 2, “The baby's arrival”. And then monthly sessions for nine months thereafter, comprising three modules preparing mother for early (2nd to 4th month postnatal), middle (5th to 7th month postnatal) and late (8th to 10th month postnatal) infancy. The timing of the sessions could be adapted according to the convenience of the intervention recipient. Each session lasted between 45 to 60 minutes. Details on the THP are provided in Figure 1 and the Thinking Healthy Manual distributed by the World Health Organizations (20).

### Baseline and Outcome Assessments

For this investigation, baseline assessments comprising of three classes of variables were used from the data set of the original trial: (a) demographic and socioeconomic variables (b) all items of the Hamilton Depression Rating Scale and (c) all items of the Brief Disability Questionnaire. All the tools were translated, adapted, and validated for the population, prior to their use in this trial.

The demographic and socioeconomic characteristics included age, education levels, income levels, parity, family structure, and empowerment. The Hamilton Depression Rating Scale (HDRS) comprised of 19 items assessing different symptoms of major depressive disorder (16, 21, 22). In addition to items mapped on the DSM-IV criteria of diagnoses, this scale also inquires about the participants' insight toward their illness, details on symptoms of physical and psychic anxiety, somatic complaints, and hypochondriasis. It also provides more information on compound symptoms in the DSM-IV diagnostic criteria. For instance, it rates symptoms of insomnia as per different stages (early, middle, and late) as well as hypersomnia. Items on problems with appetite are classified into loss of appetite and hyperphagia, as opposed to the DSM-IV. It has shown excellent validity and reliability in the screening for depression and measuring treatment response (22). All items on this scale are measured on a 4-point Likert scale. The third section comprised of the Brief Disability Questionnaire (BDQ) which measures disability among psychiatric populations. It measures dysfunction in daily physical and psychosocial functioning (16). It comprises of 8 items, recording responses on a 3-point Likert scale, ranging from '0' (not at all impaired) to '2' (moderately or definitively impaired). It has previously been found to be valid and reliable for use in the Pakistani population.

### Statistical Analysis

All data were analyzed in SPSS (v. 27). Quantitative variables were described as mean (SD) and categorical as frequencies (%). Frequencies and percentages were presented for all the symptoms records on the HDRS. Frequencies (%) were calculated for different symptom profile after breaking them into two classes: (a) similar profiles of depressive symptom (b) Disjointed symptom profiles presenting a unique symptom combination by one individual only and (c) range of symptoms endorsed in different symptom combinations.

Thereafter, we conducted exploratory factor analyses (EFA) using alpha factoring and oblique rotation (direct oblimin), to delineate the factor structure of the HDRS. Kaiser-Meyer-Olkin measure of sampling adequacy and Bartlett's test of sphericity was conducted to assess the adequacy of sample size prior to running the EFA. Suitability of items to retain in the resulting pattern matrix were decided based on communalities ≥0.20 and factor loadings ≥0.40. Number of factors to retain were judged based on Eigen value ≥1 and Cattell's Scree plot and interpretability criterion. The resulting factors were subjectively named based on predominant symptomatology captured by each factor. The resulting factors were then fed into a two-step clustering model, to cluster study participants based on their depressive symptomatology. Study participants were clustered based on Akaike's Information Criterion based on log-likelihood distance measure. Cluster quality was parsimonious at Silhouette measure of cohesion and separation >0.20.

Thereafter, to investigate the relationship between symptom clusters and treatment effectiveness in reducing depressive symptom severity and disability scores, we performed two linear mixed models analyses separately with HDRS scores and BDQ scores as dependent variables. These models were run with maximum likelihood method, and random effects for union councils of participants, to account for clustering. In both models, treatment condition and symptoms clusters of perinatal depression were added as fixed effects, with two-way interactions between both. Further decision for adding suitable covariates and choice between adding a fixed or a random intercept was based on significant improvements in 2 log-likelihood criteria.

The results of linear mixed effects models were difficult to interpret in a clinical context. Therefore, we also presented improvements in rates of perinatal depressive disorder across the four subtypes of PND. These results were presented as risk ratios for easier interpretation, and Chi-square test of association to assess statistical significance.

## Results

### Baseline Characteristics

A total of 903 pregnant women in their third trimester participated in baseline interviews. The respondents had a mean age of 26.74 (5.11), with a higher proportion being non-educated or educated till primary class. A total of 478 (52.9%) were financially empowered, with 530 (58.7%) living in extended household systems. Majority of the study participants were rated by LHWs from the poorest to lower middle-income households. Mean disability scores were 8.21 (2.69) and depression scores were 14.63 (4.09) (Table 1).

**Table 1:** Demographics and baseline characteristics of study participants (*n* = 903).

**Variable**	**Subgroup**	**Mean (SD)**	**Frequency (%)**
Age		26.74 (5.11)	
Schooling	0		374 (41.42%)
	1–6 (primary)		275 (30.45%)
	7–10 (Secondary)		221 (24.47%)
	>10		33 (3.65%)
Financially empowered	No		425 (47.1%)
	Yes		478 (52.9%)
Mean depression score		14.63 (4.09)	
Mean disability score		8.21 (2.69)	
Number of children		2.24 (1.80)	
Extended/joint family	No		373 (41.3%)
	Yes		530 (58.7%)
Health workers' rating of socioeconomic status	1 (richest)		12 (13.29%)
	2		81 (8.97%)
	3		343 (37.98%)
	4		270 (29.90%)
	5 (Poorest)		197 (21.82%)
Endorsed symptoms of perinatal depression	Depressed mood	2.24 (0.70)	900 (99.70%)
	Insight	0.13 (0.35)	118 (13.1%)
	Anhedonia	2.27 (0.65)	899 (99.6%)
	Loss of appetite	1.02 (0.72)	675 (74.8%)
	Hyperphagia	0.03 (0.24)	20 (2.2%)
	Loss of weight	0.27 (0.50)	225 (24.90%)
	Weight gain	0.02 (0.15)	15 (1.7%)
	Early insomnia	0.94 (0.79)	591 (65.4%)
	Middle insomnia	0.90 (0.82)	551 (61.0%)
	Late insomnia	0.49 (0.74)	313 (34.7%)
	Hypersomnia	0.12 (0.45)	62 (6.9%)
	Retardation	0.74 (0.54)	621 (68.80%)
	Agitation	0.06 (0.26)	44 (4.90%)
	Somatic anxiety	1.00 (0.87)	572 (63.3%)
	Somatic symptoms	1.62 (0.52)	891 (98.7%)
	Hypochondriasis	0.20 (0.49)	149 (16.50%)
	Guilt	0.37 (0.53)	308 (34.10%)
	Psychic anxiety	1.83 (0.87)	817 (90.50%)
	Suicide	0.37 (0.68)	230 (25.50%)
Number of symptoms endorsed	1 to 3	1 (0.1%)	
	4 to 5	27 (3.0%)	
	6 to 10	682 (75.5%)	
	> 10	193 (21.4%)	
Unique number of symptom profiles		365	
Disjoint combinations		237 (64.93%)	

### Heterogeneous Symptom Profiles

Almost all participants reported experiencing core symptoms of depression: depressed mood (900, 99.70%) and anhedonia (899, 99.60%). In addition to core symptoms of depression, comorbid symptoms of somatic anxiety (572, 63.30%), psychic anxiety (817, 90.50%) and somatic symptoms (891, 98.70%) were highly prevalent. Psychomotor retardation was reported by a higher proportion of respondents (621, 68.80%) than agitation (4.90%). Physical manifestations such as loss of appetite were reported by 675 (74.8%), loss of weight (225, 24.90%). Symptoms of insomnia were reported as early (591, 65.4%), middle 551 (61.0%) and late (313, 34.7%). Atypical symptoms of hyperphagia were reported by 20 (2.2%), weight gain (15, 1.7%), and hypersomnia (62, 6.9%). Suicidal ideation was reported by a quarter (25.50%) of the participants (Table 1).

Out of 19 symptoms recorded on the HDRS, most of the participants reported having 6 to 10 symptoms of depression. Less than a quarter (21.4%) reported having over ten symptoms while the rest reported between 1 to 9 symptoms. These symptoms clustered together into 365 different combinations, where 237 (64.93%) of the combinations were disjointed; reported by only one individual in the study sample. Almost all the symptom clusters had depressed mood and anhedonia as the overlapping symptom (Table 1).

### Clinical Phenotypic Clusters of PND

Two successive EFA were run before reaching an interpretable factor structure. In the first model, symptoms pertaining to agitation, hypersomnia, hyperphagia, weight gain, and guilt and insight were removed due to poor inter-item correlation and weak communalities. In the second EFA, a total of thirteen symptoms were retained in the factor analytic model, yielding adequate KMO measures of sampling adequacy (0.782) and significant Bartlett's test of sphericity. All communalities were adequate (≥0.20) except for loss of appetite (0.19), and hypochondriasis (0.19). Assessment of Eigen values and visualization of Scree plot suggested a three-factor solution for the HDRS, explaining 54.68% of the variance in HDRS (Supplementary Figure 1).

The three factors were named as: (a) core emotional symptoms representing symptoms of depressed mood, anhedonia, loss of appetite, somatic anxiety, and psychic anxiety. (b) somatic problems comprising of loss of weight, retardation, somatic symptoms, hypochondriasis, and suicidal ideation. And (c) sleep problems: insomnia during early, middle, and late night (Table 2). All these factors yielded Cronbach's Alpha > 0.60.

**Table 2:** Communalities and factor loadings for HDRS scale.

**Symptoms**		**Factor**		
	**Communalities**	**Somatic**	**Core emotional**	**Sleep**
		**symptoms**	**symptoms**	**problems**
Depressed mood	0.436		0.577	
Anhedonia	0.549		0.714	
Loss of appetite	0.191		0.411	
Loss of weight	0.423	0.552		
Early insomnia	0.449			0.639
Middle insomnia	0.635			0.800
Late insomnia	0.661			0.484
Retardation	0.221	0.460		
Somatic anxiety	0.735		0.723	
Somatic symptoms	0.260	0.380		
Hypochondriasis	0.188	0.426		
Psychic anxiety	0.386		0.573	
Suicidal ideation	0.303	0.568		

A two-step clustering algorithm clustered the study participants into four clusters based on above symptoms. In addition to the factors, we also introduced an additional variable for presence of any of the atypical symptoms (hypersomnia, hyperphagia and weight gain) which were endorsed by 88 (9.7%) of the participants. These did not load previously on the EFA, primarily due to their heterogeneous nature when compared with other symptoms. The two-step cluster model revealed a fair quality four cluster solution, with a silhouette measure of cohesion and separation at 0.5. Ratio of the largest to smallest cluster was 4.28.

**Figure 2:**
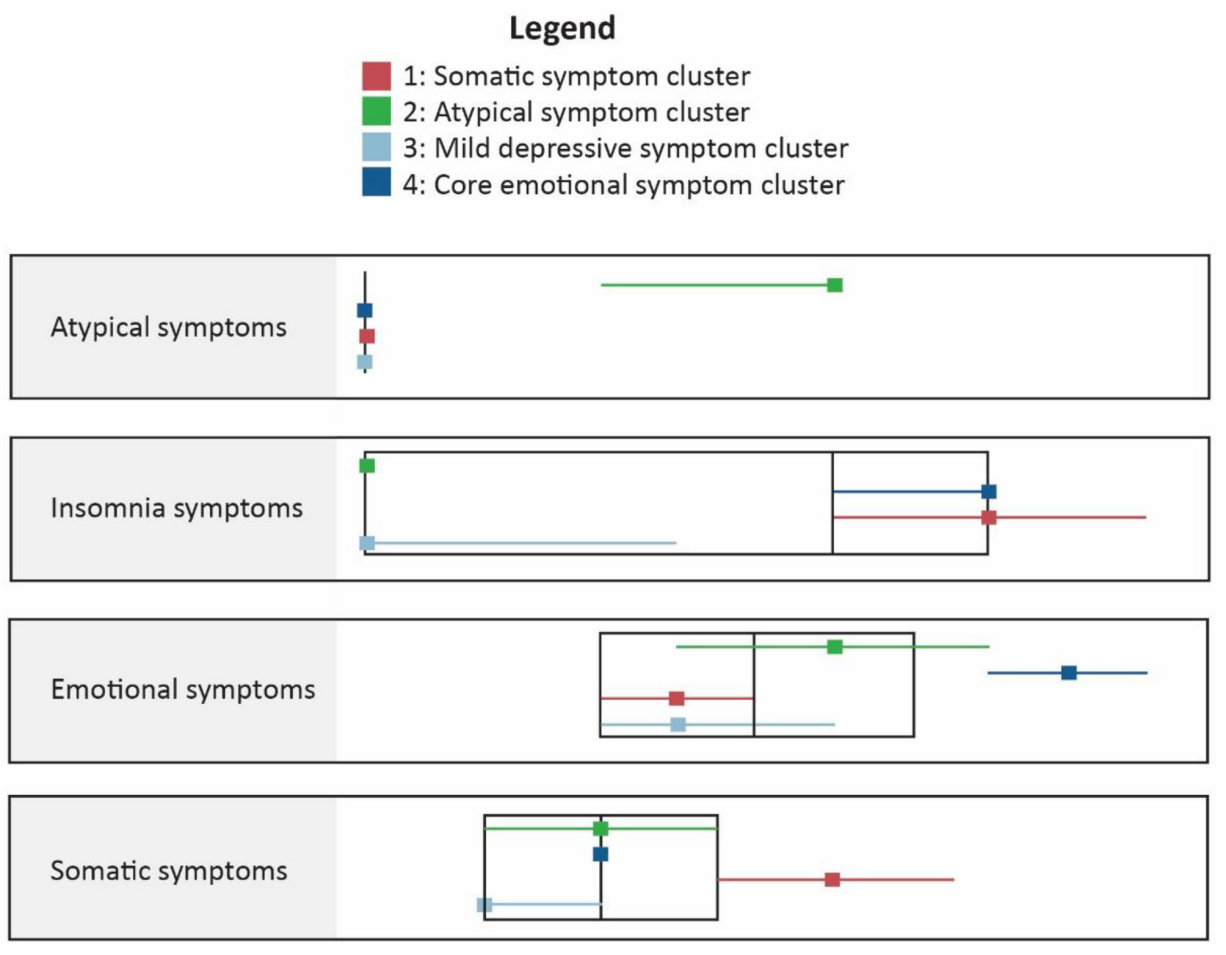
Comparison of clusters on different symptom dimensions in two step clustering analysis.

Cluster 1 (atypical depression) comprised of participants with highest proportion of atypical symptoms and mild core emotional and somatic symptoms. Cluster 2 (Somatic symptoms) reported higher scores on somatic symptoms and insomnia symptoms. Cluster 3 (mild depression) reported mild symptoms overall while the fourth cluster (mixed anxiety and depression) reported severe core emotional symptoms (Figure 2 and Supplementary Figure 2).

### Effectiveness of the Thinking Healthy Programme

**Figure 3:**
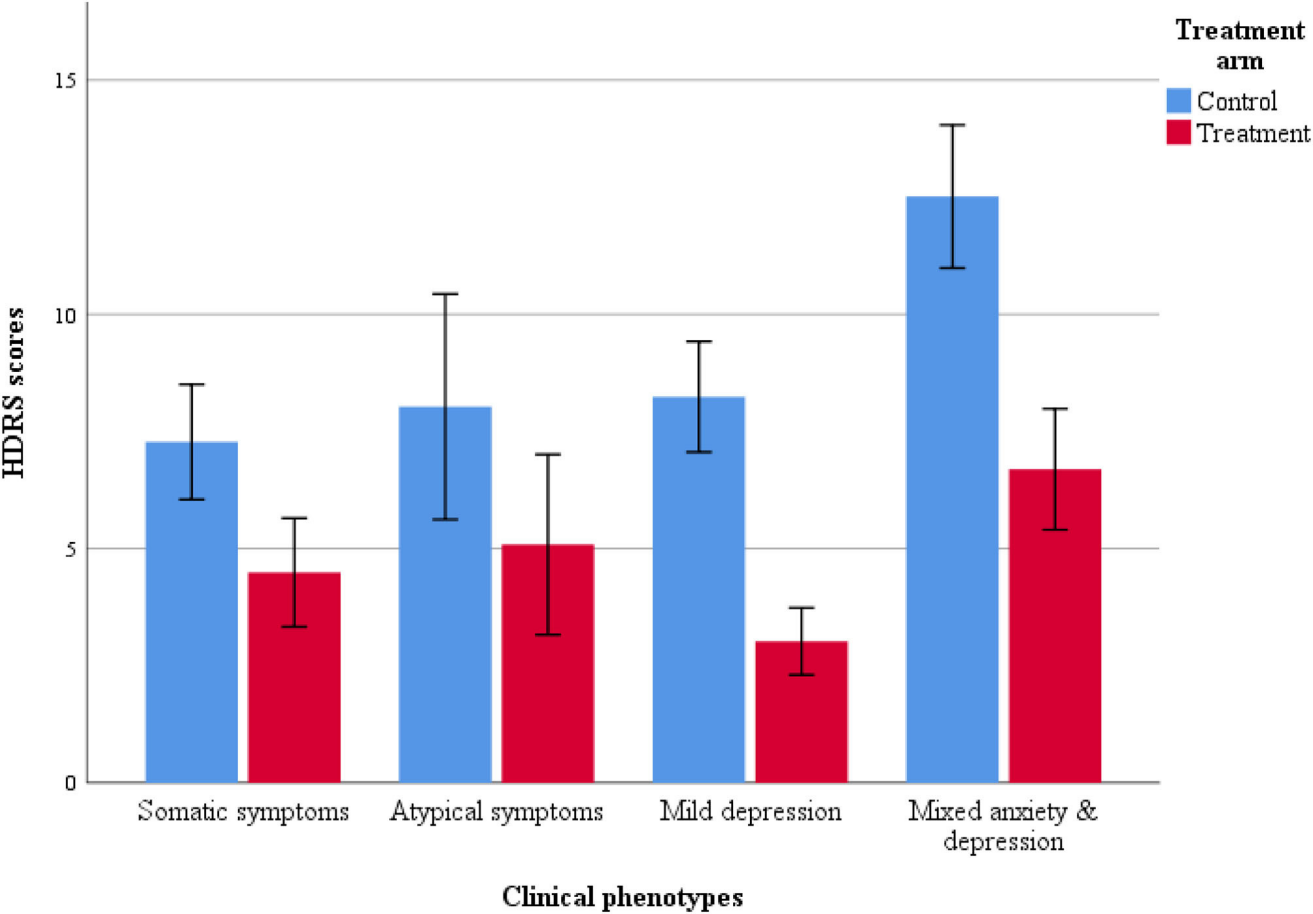
Bar plot for HDRS scores in treatment arms.

Linear mixed effects models revealed significant differences in depression severity among participants with different symptom profiles (Table 3, Figure 3). Pairwise comparisons using Bonferroni correction revealed that mixed anxiety and depression symptom cluster reported higher HDRS scores than their counterparts. The rest of the subtypes did not differ significantly on HDRS scores (Supplementary Table 1). Treatment condition x subtype interaction was also statistically significant (Supplementary Figure 3). However, fixed effects were only significant for the somatic subtype of PND. Addition of additional covariates did not lead to any significant changes in the BIC and 2-LL, however, these are presented as Supplementary Tables 2, 3.

**Table 3:** Linear fixed effects models for severity of depressive symptoms.

**Parameter**	**B**	**S.E**.	**df**	**t**	**P**	**95% Confidence interval**	
						**Lower bound**	**Upper bound**
Intercept	12.513	0.746	818.000	16.773	<0.001	11.049	13.977
Somatic depression	−5.239	0.920	818.000	−5.695	<0.001	−7.045	−3.434
Atypical depression	−4.485	1.316	818.000	−3.409	0.001	−7.068	−1.903
Mild depression	−4.274	0.924	818.000	−4.624	<0.001	−6.088	−2.459
Mixed anxiety-depression	Reference group						
Treatment arm (Tx Arm)	−5.821	1.011	818.000	−5.760	<0.001	−7.805	−3.837
Somatic depression x Tx Arm	3.036	1.271	818.000	2.389	0.017	0.541	5.531
Atypical depression x Tx Arm	2.879	1.845	818.000	1.560	0.119	−0.743	6.501
Mild depression x Tx Arm	0.601	1.263	818.000	0.476	0.634	−1.878	3.080
Mixed anxiety-depression x Tx Arm	Reference group						

**Figure 4:**
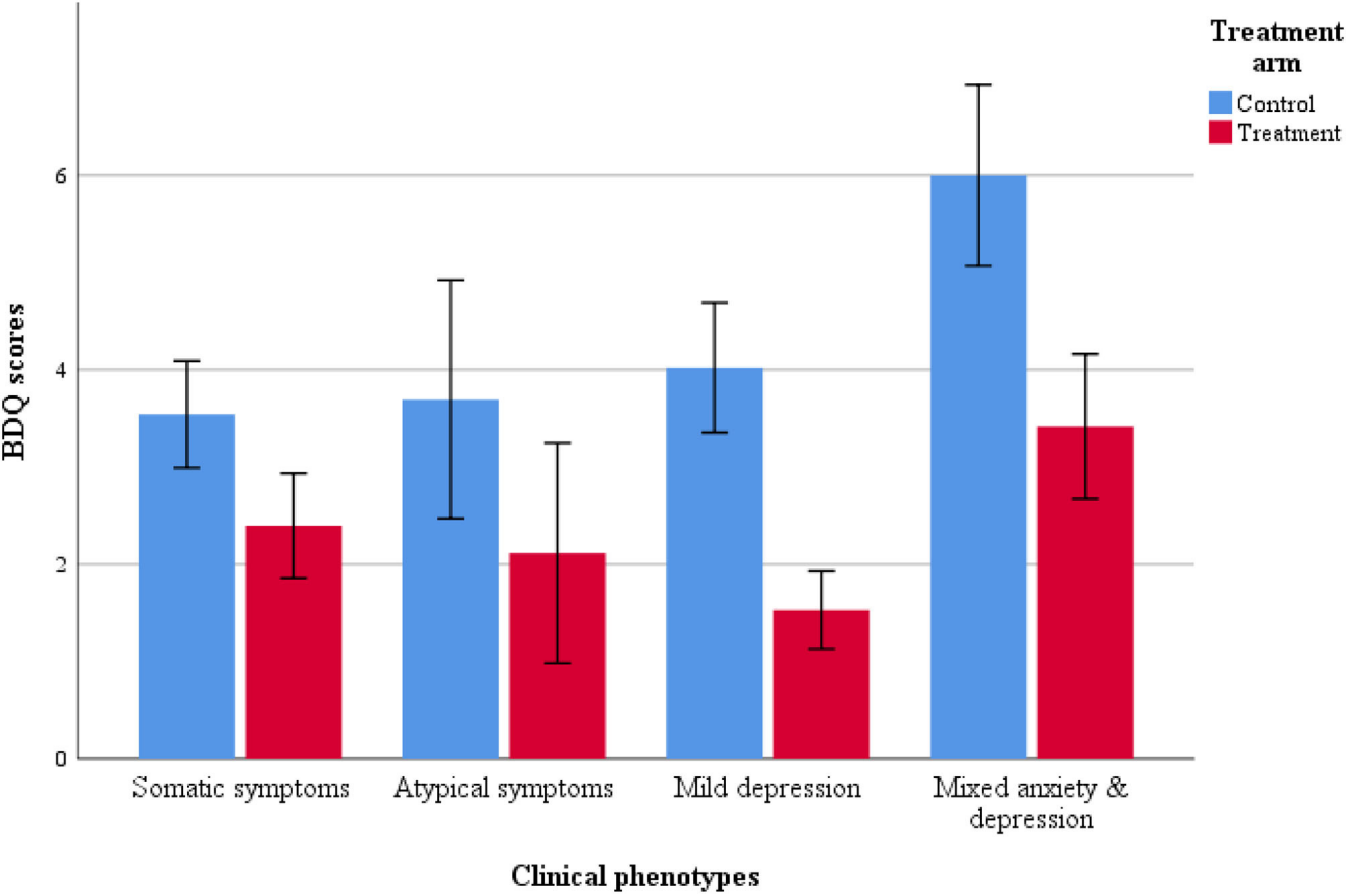
Bar plot for disability scores in treatment arms.

Similar trends were noted for the disability (BDQ) scores, participants reporting mixed anxiety and depression reported least improvement in disability when compared with other symptom clusters (Table 4 and Figure 4). However, the treatment x subtype interaction was non-significant.

**Table 4:** Linear fixed effects models for severity of disability.

**Parameter**	**B**	**S.E**.	**df**	**t**	**P**	**95% Confidence interval**	
						**Lower bound**	**Upper bound**
Intercept	6.000	0.392	818.000	15.321	<0.001	5.231	6.769
Somatic depression	−2.459	0.483	818.000	−5.092	<0.001	−3.407	−1.511
Atypical depression	−2.306	0.691	818.000	−3.338	0.001	−3.661	−0.950
Mild depression	−1.979	0.485	818.000	−4.078	<0.001	−2.931	−1.026
Mixed anxiety-depression	Reference						
Treatment arm (Tx Arm)	−2.582	0.531	818.000	−4.868	<0.001	−3.624	−1.541
Somatic depression x Tx Arm	1.437	0.667	818.000	2.154	0.032	0.127	2.747
Atypical depression x Tx Arm	1.002	0.969	818.000	1.035	0.301	−0.899	2.903
Mild depression x Tx Arm	0.091	0.663	818.000	0.137	0.891	−1.211	1.392
Mixed anxiety-depression x Tx Arm	Reference						

As per clinical assessments, all clinical phenotypes responded well to the THP programme. Compared to their counterparts in the control group, mothers with mild depression in the treatment group yielded lowest risk ratios 0.24 (95% CI: 0.15 to 0.37), followed by mothers with anxiety-depression 0.50 (95% CI: 0.37 to 0.68), atypical depression 0.51 (95% CI: 0.27 to 0.99) and somatic depression 0.59 (95% CI: 0.42 to 0.83).

## Discussion

This study presents evidence pertaining to the extent of heterogeneity in perinatal depressive symptoms in a sample of women in rural Pakistan. We found 365 different combination of symptom fulfilling DSM criteria of perinatal depression among 903 Pakistani women. Four distinct phenotypes of PND were revealed, with different clinical characteristics. The THP intervention was found to be effective in improving severity of symptoms and disability across the identified subtypes of PND.

This investigation reports a high extent of heterogeneity in perinatal depressive symptoms among diagnosed cases. Previous literature on heterogeneity of depression has focused solely on major depressive disorder in the general population (15, 23–27). Therefore, this investigation substantiates previous work by providing relevant data pertaining to PND. It highlights the shortcomings associated with the polythetic nature of psychiatric diagnoses where individuals diagnosed with similar disorder may have completely non-overlapping symptoms. This assumption is problematic because variable symptom profiles point to a single diagnosis as a latent entity, as posited in psychometrics (13). This position and underappreciation of within disorder heterogeneity, although seemingly plausible, has impeded progress in etiological and biological understanding of psychiatric diagnoses including perinatal depression (28–30). Our findings are corroborated by previous literature on major depressive disorder, which has shown this condition to be highly heterogeneous, yielding as many as 1,030 unique symptom profiles in secondary analyses of large scale datasets (27, 31).

Our analyses reveal three important insights pertaining to the polythetic nature of perinatal depression diagnosed as a set of few symptoms (including compound symptoms). Firstly, it challenges the assumption that each symptom is equally distressful to the patient. Secondly, it scrutinizes the DSM/ICD approach of presenting problems with sleep, appetite, and weight changes as compound symptoms. And lastly, that the symptoms qualifying the DSM criteria of perinatal depression warrant further scrutiny. Our model shows that even non-DSM symptoms such as physical and psychic anxiety and hypochondriasis were highly prevalent in our sample. And that these symptoms may sometimes be more distressful to the patient than the classical DSM symptoms. Therefore, a clinical staging approach rooted in a transdiagnostic framework should take precedence over traditional categorical diagnoses, at least in epidemiological studies. These findings have previously been corroborated by several studies, albeit focused on major depressive disorders, which emphasize that the consideration should be given toward study of individual symptoms rather than depending on *sum-scores* (15, 23), including the symptoms not covered by the DSM criteria (32) such as anxiety, aggression and irritability (33, 34).

More recently, with the advent of network psychometrics and mixture and cluster modeling approaches, empirical evidence has come into light, regarding the shortcomings associated with the covert within disorder heterogeneity and homogeneity and symptom overlap across different mental disorders (6, 7, 35). Most of the literature studying heterogeneity in perinatal depression have focused on either studying longitudinal trajectories of depression or classification based on severity of symptoms (6, 7). The study of longitudinal trajectories of PND have led to our understanding of variation in severity of depression according to onset during peripartum period, or nature of trajectory as subclinical, stable, chronic or remitting and relapsing (7).

However, only a few studies have considered individual symptoms leading to variation in clinical phenotypes of PND. Two studies of note explored PND using network analyses to emphasize the study of symptoms of depression because different clinical profiles of women with PND may point out to varying biological etiology. And treating all women with a similar paint brush of a categorical diagnosis may mask the underlying biological processes, and hamper treatment. In this context, Santos Jr. et al. (36) reported that cortisol levels among women with perinatal depression yield an association with positive mood and social support and negative with sadness and crying. Another study by Phua et al. emphasized the consideration of perinatal anxiety symptoms which emerged as a bridging symptom while studying depressive-anxiety network structures among Singaporean women. They also indicated that anxiety symptoms may precede development of maternal depression (8).

Similar to our study, the study of symptom clusters was taken up by an international perinatal psychiatry consortium *Postpartum Depression: Action Toward Causes and Treatment (PACT)* (37, 38). Using the Edinburgh Postnatal Depression Scale, three symptom dimensions were identified including depressed mood, anxiety and anhedonia, leading to five distinct clusters of women with PND. These clusters included “severe anxious depression, moderate anxious depression, anxious anhedonia, pure anhedonia, and resolved depression”. These subtypes differed in symptom quality, time of onset, with dimensions of anxiety and anhedonia associated with high severity and postpartum onset. Latent class analyses using the same dataset revealed three subclasses based on severity scores, with varying risk factors, resulting complications and prognosis (37, 38).

Considering the above, it is necessary to tailor interventions according to heterogeneous symptom profiles, to achieve optimum effectiveness. The *Postpartum Depression: Action Toward Causes and Treatment (PACT) Consortium* also emphasizes this need for development of personalized interventions, especially considering lower remission rates to currently available pharmacological therapies for PND (39). Cognitive behavioral therapy (CBT) as compared to other high intensity psychotherapies is evidence based and has shown good effectiveness in PND (40). Although previous research has studied effectiveness of psychological interventions and to some extent pharmacological interventions, none has acknowledged heterogeneity in this condition. Thus, there is an increasing evidence for heterogeneity in PND, and the need for tailoring of treatment for individuals. However, for most of the mental health systems in the low- and middle-income countries, this would translate to higher costs and human resource utilization. This is a considerable barrier in uptake of treatments in these regions, which are already under resourced and struggle with provision of mental health care to masses.

To bridge this evidence gap, we provide evidence for a multicomponent CBT based intervention the *Thinking Healthy Programme (THP*, for heterogeneous presentations of the PND. We hypothesized that the THP due to its multicomponent nature (20) would prove to be effective in achieving remission for different clinical phenotypes of PND. Indeed, THP was found to be effective in all the clinical phenotypes of PND in this study. The present investigation thus, strengthens evidence base for THP, proving its utility in achieving clinical and cost effective mental healthcare, especially in resource constrained areas (41). The inclusion of the THP in mhGAP programme by the WHO, provides an impetus for scale up of this programme in LMICs.

## Strengths and Limitations

This study has several strengths. It is based on a large randomly selected study sample which improves its generalizability to rural Pakistan. The participants in this study were diagnosed clinically for PND, therefore, it overcomes limitations pertaining to recall bias associated with use of psychometric scales. However, the results of this study should be generalized with caution because the symptom profiles reported in this study may differ cross-culturally.

An important limitation of this study was the lack of information pertaining to prior diagnoses of major depressive disorder. This information is immensely helpful in categorizing postpartum depression as recurrent or continuation of a previous episode of major depression. This was primarily because this intervention was conducted in a rural subdistrict, which at the time had poor awareness regarding mental illnesses and little to no access to either primary or specialized mental health care. Therefore, it is likely most of the study sample may not have been aware of their diagnoses of major depressive disorder. Inquiring about *covert* depressive mood symptoms prior to the study may have introduced significant recall bias. Moreover, the findings may have been questionable due to validity and reliability issues when labeling a participant with major depressive disorder retrospectively.

Another concern pertains to the factorial invariance of the HDRS, which has often been raised in depression research. This issue was highlighted by Bagby et al. who reviewed evidence pertaining to psychometric properties of the HDRS (42). They noted that HDRS yielded inconsistent factor structures across different populations and studies. Authors reported as few as 2 and as many as 8 subscales of HDRS, with inconsistent item level factor structures and loading values. They concluded that HDRS was thus, conceptually flawed, and newer tools were needed based on the DSM and contemporary definitions of depression. Unfortunately, all the reviewed studies were performed among populations other than the perinatal ones. However, we opine that this factorial invariance of HDRS across different populations merely points to the covert heterogeneity of major depressive disorder and the need for studying different subtypes of depression rather than treating it as a single syndromic construct (13).

### Implication for Future Research and Implementation

This study, comprising of a largely descriptive evidence of heterogeneity in PND, should inspire further empirical work in this domain. Based on our results, we emphasize the adoption of a transdiagnostic framework in etiological and intervention research pertaining to PND. PND when visualized in a transdiagnostic framework should overcome limits related to study of a subset of symptoms of PND (43). We recommend that interventions should be designed in a transdiagnostic framework to account for comorbid symptoms of anxiety, hypochondriasis, and somatic complaints. We also recommend that future research should study clustering of symptoms of PND to inform The Hierarchical Taxonomy of Psychopathology (HiTOP) and Research Domain Criteria (rDOC) frameworks (44, 45). It is also important to study PND from a network perspective to delineate important symptoms as targets for interventions and overlap with other DSM disorders (29). From the perspective of psychometrics, it is important to consider development and deployment of scales that account for prevalent symptoms comorbid with PND for screening as well as measurement of response to intervention (46).

## Data Availability Statement

The raw data supporting the conclusions of this article will be made available by the authors, without undue reservation.

## Ethics Statement

The studies involving human participants were reviewed and approved by Ethical approval for the original randomized controlled trial was granted by the University of Manchester, UK and the Institute of Psychiatry, Rawalpindi, Pakistan. The present secondary analyses, however, were exempt from ethical review. The patients/participants provided their written informed consent to participate in this study.

## Disclosure

The preliminary findings from this study were presented at the Annual Congress of the European Psychiatric Association. This manuscript is to be submitted as partial fulfilment for a Ph.D. degree in Psychiatry at the University of Liverpool, Liverpool, UK.

## Author Contributions

AW conceived the idea, conducted the secondary analyses, and wrote the first draft of the manuscript. AR led the original project, supervised the current analyses, and extensively edited the first draft of the manuscript. All authors approved the manuscript.

## Conflict of Interest

The authors declare that the research was conducted in the absence of any commercial or financial relationships that could be construed as a potential conflict of interest.

## Publisher's Note

All claims expressed in this article are solely those of the authors and do not necessarily represent those of their affiliated organizations, or those of the publisher, the editors and the reviewers. Any product that may be evaluated in this article, or claim that may be made by its manufacturer, is not guaranteed or endorsed by the publisher.
