# Supplementary material for: Does One Treatment Fit All? Effectiveness of a Multicomponent Cognitive Behavioral Therapy Program in Data-Driven Subtypes of Perinatal Depression
Source: Front Psychiatry. 2021 Nov 17;12:736790. doi: 10.3389/fpsyt.2021.736790 (PMC8635695; doi:10.3389/fpsyt.2021.736790)

Supplementary table 1: Pairwise comparisons for subtypes of perinatal depression as per depressive symptoms severity

| (I) Clusters | (J) Clusters | Mean Difference (I-J) | Std. Error | df | p-value | 95% Confidence Interval for Difference^a^ | |
| --- | --- | --- | --- | --- | --- | --- | --- |
|  |  |  |  |  |  | Lower Bound | Upper Bound |
| Somatic symptoms | Atypical symptoms | -1.093 | .856 | 818 | 1.000 | -3.357 | 1.172 |
|  | Mild depression | -1.525 | .655 | 818.000 | .121 | -3.258 | .207 |
|  | Mixed anxiety & depression | -2.412* | .682 | 818.000 | .003 | -4.215 | -.609 |
| Atypical symptoms | Somatic symptoms | 1.093 | .856 | 818 | 1.000 | -1.172 | 3.357 |
|  | Mild depression | -.433 | .897 | 818.000 | 1.000 | -2.806 | 1.941 |
|  | Mixed anxiety & depression | -1.319 | .978 | 818.000 | 1.000 | -3.905 | 1.266 |
| Mild depression | Somatic symptoms | 1.525 | .655 | 818.000 | .121 | -.207 | 3.258 |
|  | Atypical symptoms | .433 | .897 | 818.000 | 1.000 | -1.941 | 2.806 |
|  | Mixed anxiety & depression | -.887 | .900 | 818.000 | 1.000 | -3.266 | 1.492 |
| Mixed anxiety & depression | Somatic symptoms | 2.412* | .682 | 818.000 | .003 | .609 | 4.215 |
|  | Atypical symptoms | 1.319 | .978 | 818.000 | 1.000 | -1.266 | 3.905 |
|  | Mild depression | .887 | .900 | 818.000 | 1.000 | -1.492 | 3.266 |
| Based on estimated marginal means | | | | | | | |
| *. The mean difference is significant at the .05 level. | | | | | | | |
| a. Adjustment for multiple comparisons: Bonferroni. | | | | | | | |

**Supplementary Table 2: MLM model for HDRS scores**

| Parameter | B | Std. Error | df | t | P-value | 95% Confidence Interval | |
| --- | --- | --- | --- | --- | --- | --- | --- |
|  |  |  |  |  |  | Lower Bound | Upper Bound |
| Intercept | 0.570 | 2.355 | 794.704 | 0.242 | 0.809 | -4.052 | 5.193 |
| Somatic depression | -1.520 | 0.763 | 336.496 | -1.991 | 0.047 | -3.022 | -0.018 |
| Atypical depression | -1.080 | 0.975 | 808.572 | -1.107 | 0.269 | -2.994 | 0.835 |
| Mild depression | -0.325 | 0.890 | 801.913 | -0.365 | 0.715 | -2.073 | 1.422 |
| Mixed anxiety and depression | Reference |  |  |  |  |  |  |
| Treatment arm | -4.112 | 0.675 | 32.694 | -6.092 | <0.001 | -5.485 | -2.738 |
| Mother's age | 0.052 | 0.046 | 800.208 | 1.132 | 0.258 | -0.038 | 0.142 |
| Maternal education | -0.105 | 0.060 | 801.086 | -1.767 | 0.078 | -0.222 | 0.012 |
| Socioeconomic class | 0.705 | 0.244 | 807.741 | 2.892 | 0.004 | 0.226 | 1.183 |
| HDRS scores at baseline | 0.363 | 0.082 | 803.271 | 4.415 | <0.001 | 0.202 | 0.524 |

**Supplementary Table 3: MLM model for BDQ scores**

| **Parameter** | **B** | **Std. Error** | **df** | **t** | **P-value** | **95% Confidence Interval** | |
| --- | --- | --- | --- | --- | --- | --- | --- |
|  |  |  |  |  |  | **Lower Bound** | **Upper Bound** |
| Intercept | 1.247 | 1.074 | 776.914 | 1.161 | 0.246 | -0.861 | 3.354 |
| Somatic depression | -1.063 | 0.385 | 233.042 | -2.761 | 0.006 | -1.821 | -0.304 |
| Atypical depression | -1.349 | 0.488 | 804.565 | -2.764 | 0.006 | -2.307 | -0.391 |
| Mild depression | -1.033 | 0.363 | 796.068 | -2.843 | 0.005 | -1.746 | -0.320 |
| Mixed anxiety and depression | Reference |  |  |  |  |  |  |
| Treatment arm | -1.791 | 0.317 | 34.180 | -5.651 | 0.000 | -2.435 | -1.147 |
| Mother's age | 0.043 | 0.024 | 805.133 | 1.789 | 0.074 | -0.004 | 0.091 |
| Maternal education | -0.053 | 0.032 | 805.644 | -1.666 | 0.096 | -0.115 | 0.009 |
| Socioeconomic class | 0.362 | 0.129 | 808.994 | 2.813 | 0.005 | 0.110 | 0.615 |
| Disability scores at baseline | 0.181 | 0.049 | 790.952 | 3.718 | 0.000 | 0.085 | 0.276 |

Supplementary Figure 1: Scree plot representing number of factors to retain for Hamilton Depression Rating Scale


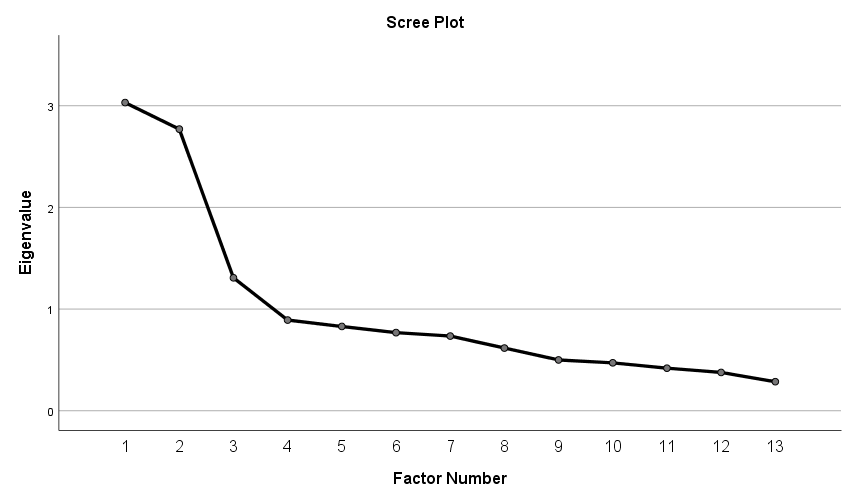


Supplementary Figure 2: Cluster comparisons for perinatal depression


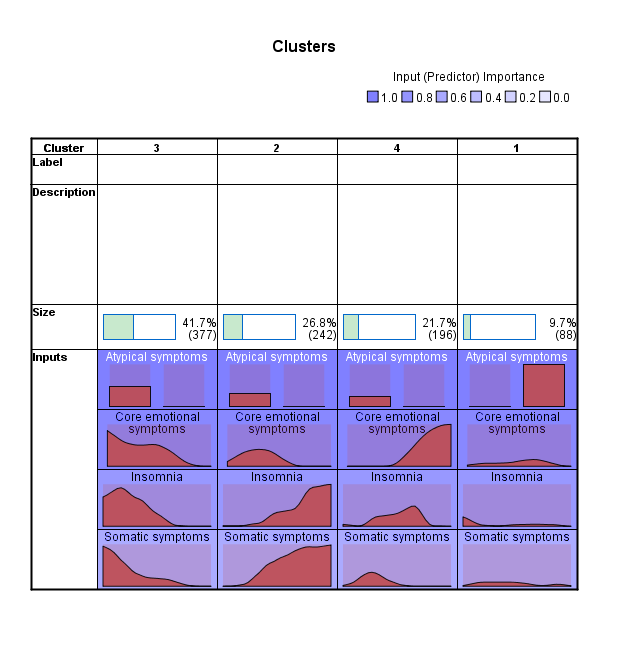

Supplement: Supplementary file 1 [file Data_Sheet_1.docx]
